# Oenothein B, a Cyclic Dimeric Ellagitannin Isolated from *Epilobium angustifolium*, Enhances IFNγ Production by Lymphocytes

**DOI:** 10.1371/journal.pone.0050546

**Published:** 2012-11-30

**Authors:** Andrew G. Ramstead, Igor A. Schepetkin, Mark T. Quinn, Mark A. Jutila

**Affiliations:** Department of Immunology and Infectious Diseases, Montana State University, Bozeman, Montana, United States of America; Hannover Medical University (MHH), Germany

## Abstract

Oenothein B is a polyphenol isolated from *Epilobium angustifolium* and other plant sources, which has been reported to exhibit immunomodulatory properties. Oenothein B is known to activate myeloid cells and induce the production of IL-1 and other cytokines. However, its effects on lymphocytes are unknown. In this report, we show that oenothein B stimulated innate lymphocytes, including bovine and human γδ T cells and NK cells, resulting in either increased CD25 and/or CD69 expression. We also demonstrate that oenothein B enhanced the production of interferon-γ (IFNγ) by bovine and human NK cells alone and in combination with interleukin-18 (IL-18), a response not observed with other commonly studied polyphenols. Furthermore, we demonstrate that oenothein B enhanced the production of IFNγ by human T cells. Since IFNγ contributes to antitumor, antibacterial, and antiviral cell responses, these data suggest an additional mechanism that could account, at least in part, for the immune enhancing properties of oenothein B.

## Introduction

Nutritional supplements have been studied over many years for their ability to treat and prevent disease, including cancer and infections. Polyphenols represent a group of plant compounds found in many supplements that have been studied extensively for their role in promoting human health. Numerous studies have focused on the antioxidant properties of polyphenols; however, the antioxidant effects of nutritional polyphenols *in vivo* are controversial [1]. In addition, there are numerous studies that demonstrate biological activity of polyphenols beyond antioxidant activity, including modulating enzyme activity [2], receptor signaling [3], and immunity [4]–[7].

Innate lymphocytes, such as NK cells and γδ T cells, play an important role in host defense against cancer and various pathogens, and enhancing the activity of these cells is an attractive option for immunotherapy [8]–[10]. Results by our group and others have shown that some nutritional supplements are useful sources of novel agonists for innate lymphocytes and that the use of these supplements may represent a novel strategy to enhance the activity of these cells [4]–[7], [11]–[12]. For example, alkylamines from tea, apples, and wine, polysaccharides from Acai fruit and *Funtumia elastica* bark, and other plant components have been shown to activate and enhance the proliferation of γδ T cells [13]–[16]. In addition, we have recently found that certain polyphenols, such as oligomeric procyanidins (OPCs) from apple peel, also stimulate innate lymphocytes, from different animals, including humans [4]. However, not all polyphenols are capable of stimulating innate lymphocytes, and the size and structure of these compounds are important for their immunomodulating properties [17], [18].

NK cells and γδ T cells provide an early source of several cytokines, including interferon-γ (IFNγ) and IL-17 [19]–[21]. The production of IFNγ by lymphocytes is important in immune defense against various tumors ad infections [22]–[24] and could provide a possible mechanism for the antibacterial, antiviral, and antitumor properties proposed for certain polyphenols. However, the induction of IFNγ by polyphenols is poorly understood or defined. In our earlier study of OPCs, we found no evidence for the induction of IFNγ in innate lymphocytes. Conversely, we have detected some IFNγ production from human PBMCs treated with oenothein B, a unique polyphenol with different structural and immunological properties than OPCs [7]. Therefore, we investigated whether oenothein B might induce IFNγ production in innate lymphocytes or, based on our earlier studies that showed OPCs can enhance responses to secondary signals, possibly prime innate lymphocytes to respond more robustly to known inducers of IFNγ, such as IL-18 [25].

Briefly, oenothein B is a dimeric, macrocyclic ellagitannin isolated from *Epilobium angustifolium*, as well as other plant sources. It has been studied for antitumor, antiviral, antibacterial, antioxidant, pro-inflammatory, and anti-inflammatory properties [7], [26]–[31]. Oenothein B has been reported to inhibit inflammatory responses by phagocytes induced by TLR agonists and other stimulants [30], [31]. However, in the absence of additional stimulation, oenothein B promotes inflammatory responses by phagocytes. In studies conducted in the early 1990’s, oenothein B was shown to reduce the growth of several tumors *in vivo* and activate macrophages, promoting the production of IL-1 [28]. Induced IL-1 production was proposed to be important in the antitumor properties of oenothein B, although this has not been directly tested. We recently showed that oenothein B induces the production of IL-1, as well as other pro-inflammatory cytokines, including IL-6 and tumor necrosis factor α (TNFα), by monocytes [7], responses not seen with OPCs. In addition, we showed that substructures of oenothein B did not stimulate phagocytes to the same extent as oenothein B [7], suggesting an important role for the complete structure in its immunological activity. To date, there are no reports on the effects of oenothein B on lymphocytes. We now show that oenothein B stimulates innate lymphocytes (γδ T cells and NK cells) and promotes their production of IFNγ. We also describe a novel priming effect of oenothein B on NK cells, leading to enhanced IFNγ production following IL-18 treatment. Finally, we describe a similar priming effect of oenothein B in response to a tumor cell line.

**Figure 1:**
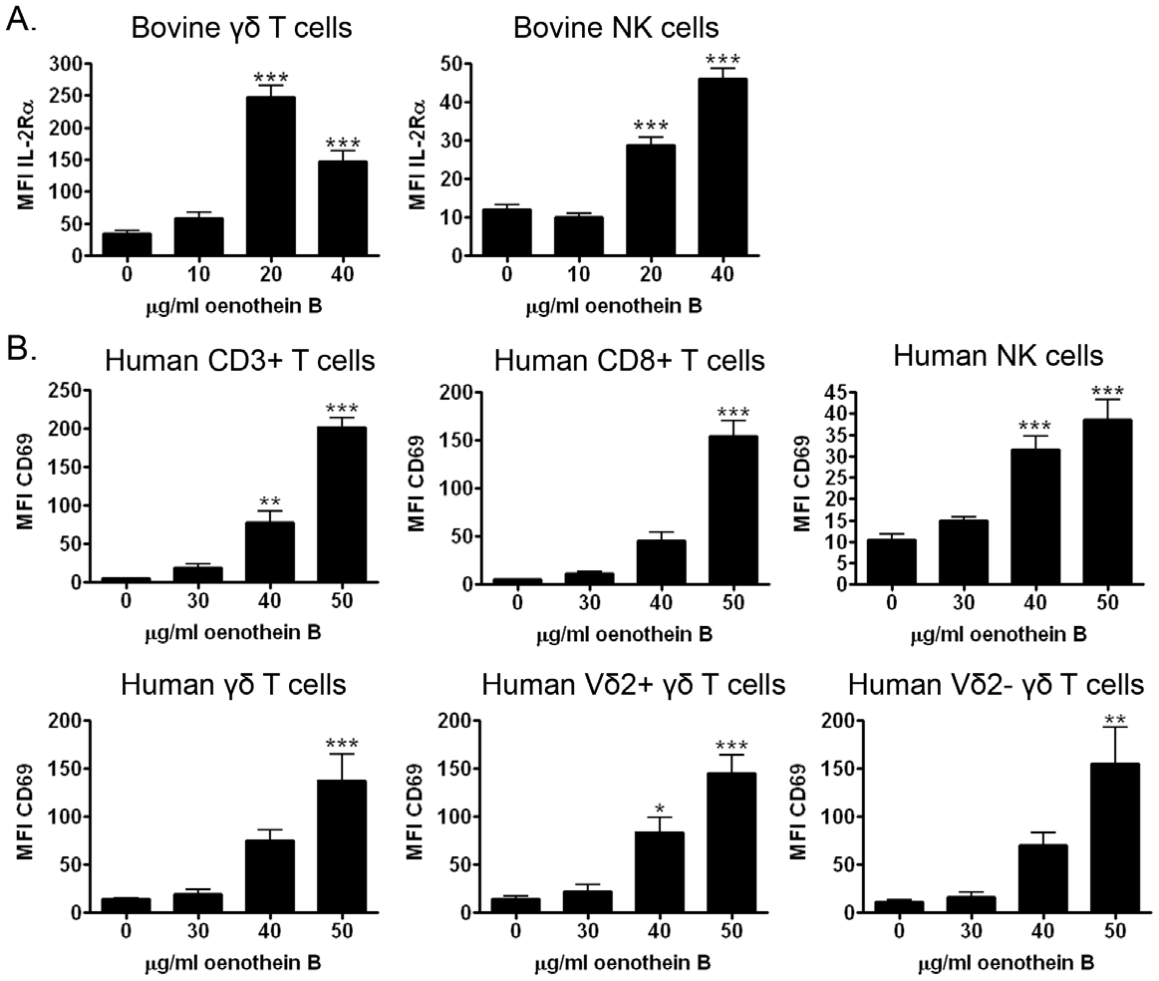
Oenothein B induces IL-2Rα or CD69 on bovine and human lymphocyte subsets. (A) Bovine PBMCs (10^5^ cells/well) were treated with the indicated concentrations of oenothein B in X-VIVO medium for 24 hrs, and IL-2Rα expression on γδ T cells and NK cells was measured by multi-color flow cytometry. NK cells were defined as non-γδ T cells that expressed CD335. The graphs represent pooled data from 3 individuals. Each treatment was analyzed in triplicate and error bars indicate SEM. Significance compared to untreated cells (0 µg/ml) was determined by One-way ANOVA with Bonferroni post-test. *p<0.05, **p<0.01, ***p<0.001 (B) Human PBMCs (10^5^ cells/well) were treated with the indicated concentrations of oenothein B in cRPMI medium for 48 hrs. CD69 expression on lymphocytes, which included CD3+ T cells, CD8+ T cells, γδ T cells, and NK cells, was then measured by flow cytometry. The graphs represent pooled data from 5 individuals. Each treatment was analyzed in triplicate and error bars indicate SEM. Significance compared to untreated cells (0 µg/ml) was determined by One-way ANOVA with Bonferroni post-test. *p<0.05, **p<0.01, ***p<0.001.

**Figure 2:**
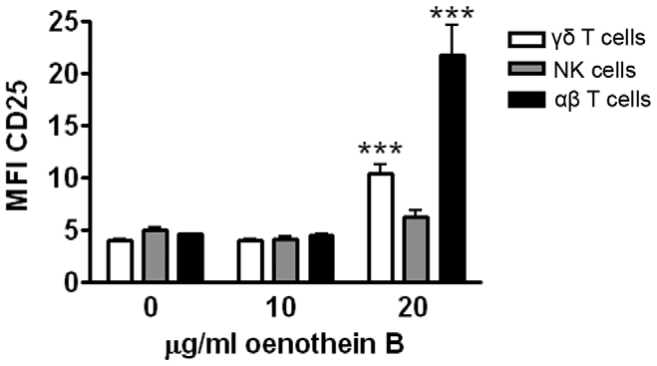
Oenothein B induces CD25 on human T cells. Human PBMCs (10^5^ cells/well) were treated with the indicated concentrations of oenothein B in X-VIVO medium for 42 hrs. CD25 expression on lymphocytes, which included γδ T cells (CD3+/γδ TCR+), NK cells (CD3−/CD56+), and αβ T cells (CD3+/γδ TCR-), was then measured by flow cytometry. The graph represents pooled data from 5 individuals. Each treatment was analyzed in duplicate and error bars indicate SEM. Significance compared to untreated cells (0 µg/ml) was determined by One-way ANOVA with Bonferroni post-test. *p<0.05, **p<0.01, ***p<0.001.

## Materials and Methods

### Ethics Statement

All animal experiments were performed in accordance with National Institutes of Health guidelines and approved by the Institutional Animal Care and Use Committee of Montana State University (protocol identification: 2009–3, 2011–61). Human subjects testing was performed in accordance with a protocol approved by the Institutional Review Board of Montana State University (approval identification: MJ032609), and written, informed consent was obtained from all individuals. No specific permits were required for the described field studies involving *E. angustifolium*. According to the Gallatin National Forest Office (Montana), collection of limited amounts of plant materials for non-commercial, educational purposes does not require a permit. All plants were collected from a National Forest and public land and no endangered or protected species were collected.

### Isolation of Oenothein B

Oenothein B was isolated and identified as described previously [7]. Briefly, fully blossomed *E. angustifolium* were collected and the dried plant material (400*g*) was extracted with 80% methanol at room temperature for 3 days. The combined extracts were concentrated, and any precipitates were removed by filtration through a 0.22-µm filter. The filtrate was lyophilized to obtain the crude extract or subjected to concentration and fractionation on a Sephadex LH-20 column (2.8 × 33 cm) using 80% methanol as an eluent. The relevant fractions were pooled and evaporated to dryness, re-chromatographed twice, and compound identification was performed by NMR and mass spectrometry, as described [7]. Purity was determined to be >95% by HPLC and mass spectrometry, as described [7]. A *Limulus* amebocyte lysate assay kit (Cambrex, East Rutherford, NJ) was used to evaluate possible endotoxin contamination in purified oenothein B. Purified oenothein B found to be free of endotoxin was stored at -80°C until used in the functional assays described below.

**Figure 3:**
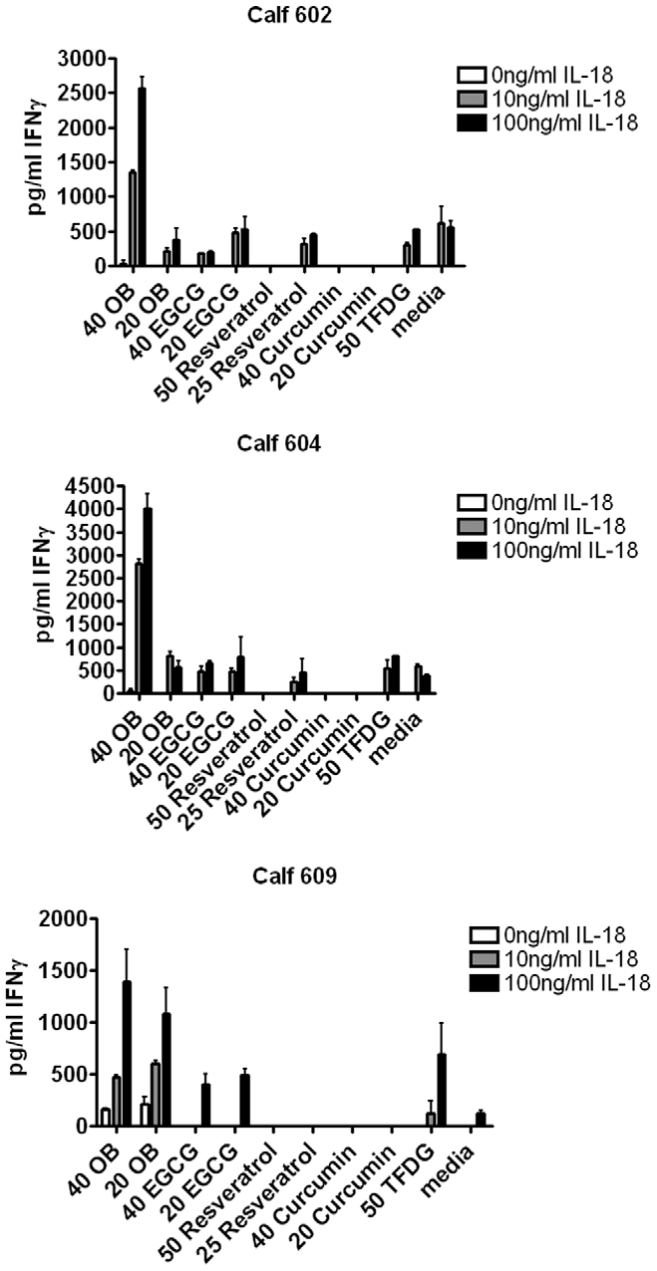
Oenothein B primes bovine PBMCs to respond to IL-18. Bovine PBMCs (10^5^ cells/well) were treated with oenothein B (40 µg/ml and 20 µg/ml), EGCG (40 µg/ml and 20 µg/ml), resveratrol (50 µg/ml and 25 µg/ml), curcumin (40 µg/ml and 20 µg/ml), theaflavin digallate (50 µg/ml), or X-VIVO medium alone for approximately 24 hrs. Cells were then washed and treated with 10 ng/ml rhu IL-18, 100 ng/ml rhu IL-18, or X-VIVO medium alone for approximately 24 hrs. After incubation, soluble IFNγ levels were measured by ELISA. The data are expressed as mean +/− SEM of three independent experiments.

### Human and Bovine Peripheral Blood Mononuclear Cell Preparations

Whole blood was collected from 1- to 3-month bull Holstein calves into sodium heparin tubes (BD Biosciences, San Jose, CA) and from healthy human adult donors with ACD solution B anticoagulant tubes (BD Biosciences). Mononuclear cells were separated from whole blood using Histopaque 1077 (Sigma-Aldrich, St. Louis, MO) or Ficoll-Paque™Premium (GE Healthcare, Piscataway, NJ) for bovine and human cells, respectively, as previously described [4] and per the manufacturer’s instructions. Additionally, bovine red blood cells were removed by hypotonic lysis after Histopaque separation.

### Flow Cytometric Analysis and Cell Sorting of Bovine and/or Human PBMCs

PBMCs were suspended in X-VIVO 15 serum-free medium or RPMI 1640 medium containing supplements and 10% FBS (cRPMI [4]). Cells were then cultured with or without oenothein B at 37°C and 10% CO_2_. Bovine cells were stained with antibodies against IL-2Rα (LCTB2A, VMRD), CD335 (AKS1, AbDSerotec), γδ TCR (GD3.8 [32]), or a bovine monocyte antigen (BN180). Human cells were stained with antibodies against CD69 (FN50, Biolegend), CD25 (M-A251, BD Pharmingen), CD3 (UCHT1, Biolegend), CD56 (MEM-188, Biolegend and CM55B, eBioscience), CD8 (HIT8a, Biolegend), γδ TCR (11F2, BD Biosciences), Vδ2 (B6, BD Pharmingen), or IL-18Rα (H44, Biolegend). All antibodies were directly labeled or indirectly labeled using goat anti-mouse FITC, PE, or APC (Jackson ImmunoResearch Laboratories, West Grove, PA). After staining, cells were analyzed using a BD Biosciences FACSCalibur with high throughput sampling (HTS).

Removal of bovine CD335+ cells, γδ T cells, and monocytes from PBMC preparations was performed using flow cytometric sorting. Briefly, bovine CD335+ cells, γδ T cells, and monocytes were stained with monoclonal antibodies (mAb) against CD335 (AbDSerotec), γδ TCR (GD3.8), and monocytes (BN180), respectively. Negative cells were then purified using a BD Biosciences FACSAria cell sorter to achieve >95% purity. As a control, unsorted bovine PBMCs were collected from the FACSAria for undepleted controls (controlling for the effects of the sorting procedure). Human NK cells were also sorted. Briefly, NK cells were isolated by staining cell preparations with CD3 (UCHT1, Biolegend) and CD56 (CM55B, eBioscience) and sorting CD3−/CD56+ cells using the FACSAria cell sorter to achieve >95% purity. After sorting, human cells were allowed to rest overnight in cRPMI with 10% FBS at 37°C and 10% CO_2_ before being used in the experiments described below.

**Figure 4:**
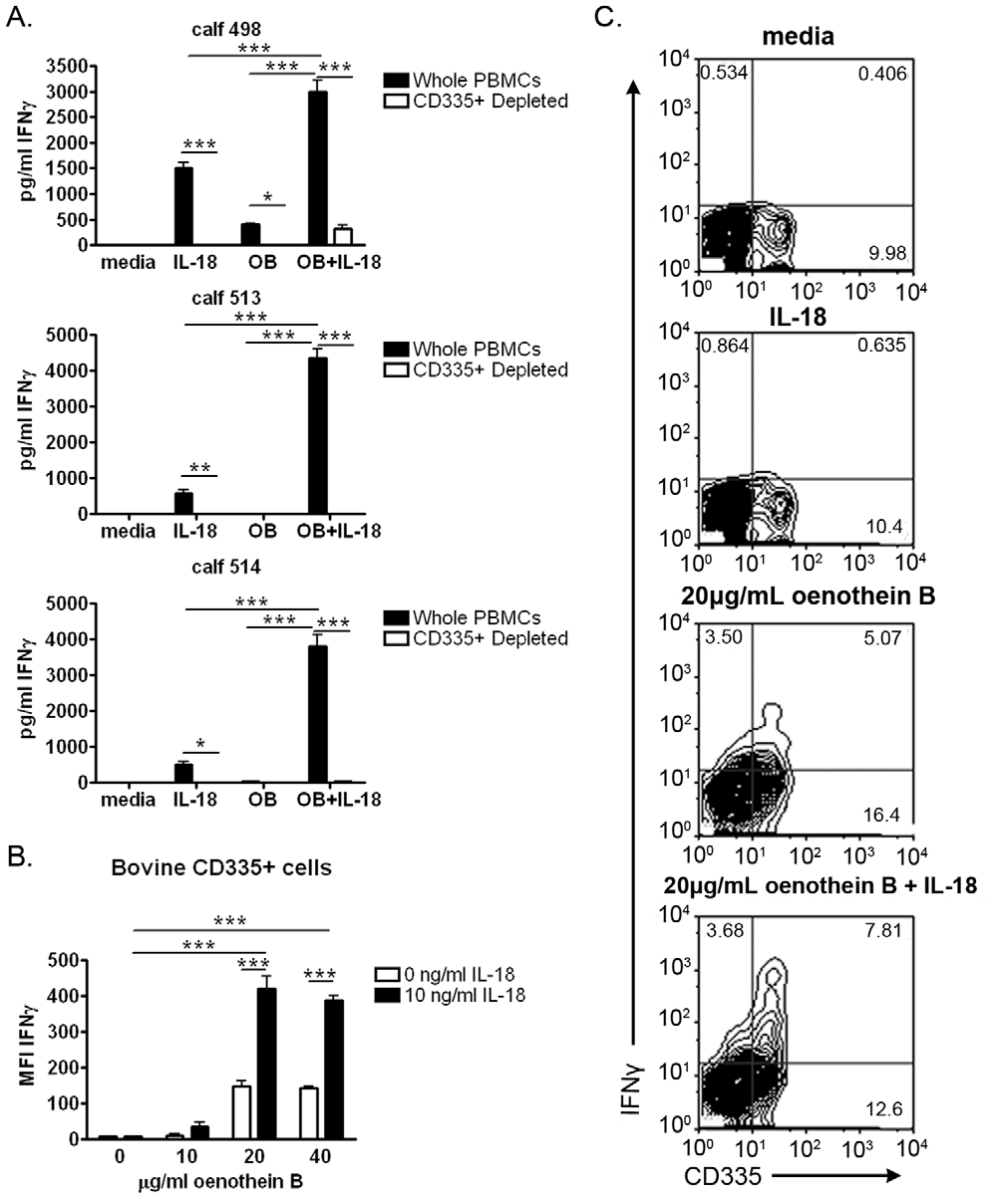
Oenothein B Primes bovine CD335+ cells to respond to IL-18. (A) Bovine PBMCs (10^5^ cells/well) were depleted of CD335+ cells and treated with 20 µg/ml oenothein B or X-VIVO medium alone for 24 hrs. Cells were then washed and treated with 10 ng/ml rhu IL-18 or medium alone for 18 hrs. After incubation, IFNγ levels in the supernatant fluids were measured by ELISA. The data are expressed as mean +/− SEM of three independent experiments comparing depleted PBMCs to un-depleted controls tested concurrently. All samples were tested in duplicate or triplicate. Statistical significance was measured by Two-way ANOVA with Bonferroni post-test. *p<0.05, **p<0.01, ***p<0.001 (B) Bovine PBMCs (10^5^ cells/well) from a new calf were treated with the indicated amounts of oenothein B or X-VIVO medium alone for 24 hrs. Cells were washed and treated with 10 ng/ml rhu IL-18 or X-VIVO medium alone for 6 hrs in the presence of brefeldin A. IFNγ production was measured by intracellular flow cytometry. The data are expressed as mean+/− SEM. All samples were tested in triplicate. Statistical significance was measured by Two-way ANOVA with Bonferroni post-test. *p<0.05, **p<0.01, ***p<0.001 (C) Representative examples of two-color flow cytometry plots comparing IFNγ staining on CD335+ cells.

**Figure 5:**
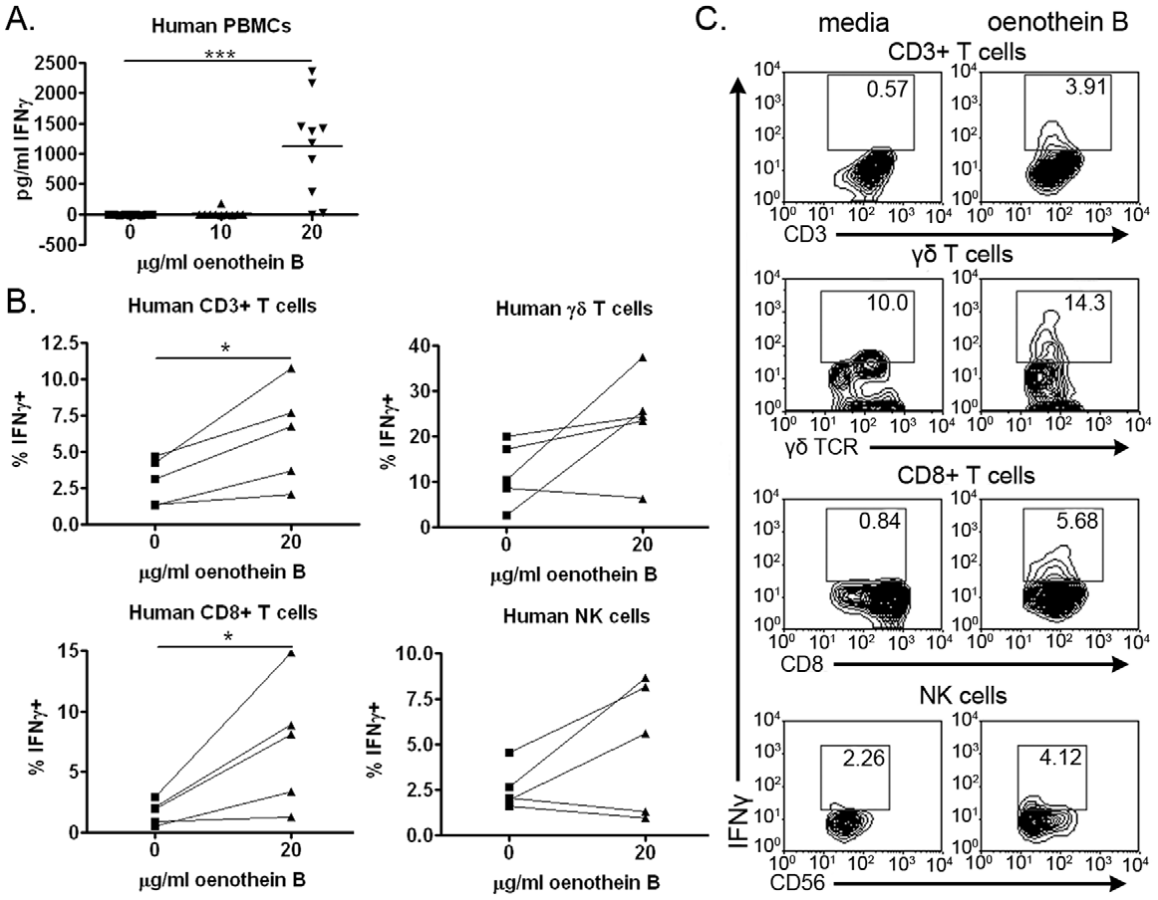
IFNγ production by human lymphocytes in response to oenothein B. (A) Human PBMCs (10^5^ cells/well) were treated with the indicated concentrations of oenothein B or X-VIVO medium alone for 48 hrs, and soluble IFNγ levels in supernatant fluids were measured by ELISA. The graph represents data from ten individuals, with each sample plated in triplicate. Statistical significance was measured by One-way ANOVA with Bonferroni post-test. *p<0.05, **p<0.01, ***p<0.001 (B) Human PBMCs (10^5^ cells/well) were treated with oenothein B or X-VIVO medium alone for 6 hrs in the presence of brefeldin A. The percent of total CD3+ T cells, γδ T cells, CD8+ T cells, and NK cells positive for IFNγ staining was then determined by flow cytometry. The graphs represent data for five individuals, with each treatment analyzed in triplicate. Statistical significance was determined by paired Student’s t-test. *p<0.05, **p<0.01, ***p<0.001 (C) Representative examples of two-color flow cytometry plots comparing IFNγ staining on oenothein B-treated and untreated human lymphocytes.

### IL-18 Activation Assays

To test for priming effects by oenothein B, bovine and human PBMCs were isolated and incubated in X-VIVO 15 medium at 37°C and 10% CO_2_ in the presence of oenothein B (0–40 µg/ml) or medium only for approximately 24 hrs (bovine cells) or 48 hrs (human cells). Cells were then washed with Dulbecco’s PBS and resuspended in X-VIVO 15 medium in the presence or absence of recombinant human (rhu) IL-18 (R&D Systems, Minneapolis, MN). A fraction of the cells were then incubated approximately 18 hrs, and the supernatant fluids were collected for IFNγ quantification by ELISA (see below). Other cells were treated with brefeldin A (eBioscience), incubated for 6 hrs, stained for intracellular IFNγ using anti-IFNγ antibodies, and analyzed by flow cytometry (see below).

Sorted human NK cells were resuspended in X-VIVO 15 medium and plated in a 96-well plate at 5×10^4^ cells/well. Cells were treated with oenothein B (20 µg/ml), rhu IL-18 (100 ng/ml), both, or medium only. Cells were incubated for 24 hrs and supernatant fluids were collected for IFNγ quantification by ELISA (see below).

### K562 Assay

K562 (chronic myelogenous leukemia) human cell line was from American Type Culture Collection (Manassas, Virginia). Human PBMCs were isolated and incubated in X-VIVO 15 medium at 37°C and 10% CO_2_ in the presence of oenothein B (20 µg/ml) or medium only for approximately 24 hrs. Cells were then washed with X-VIVO 15 and subsequently cultured in X-VIVO 15 in the presence or absence of K562 target cells. To measure soluble IFNγ, cells were co-cultured for 42 hours at 37°C and 10% CO_2_. Supernatant fluids were then collected for IFNγ quantification by ELISA (see below). To measure intracellular IFNγ, cells were co-cultured for 24 hours at 37°C and 10% CO_2_ with brefeldin A added for the final 6 hours. IFNγ quantification was then performed by flow cytometry (see below).

### Measurement of IFNγ

Enzyme-linked immunosorbent assays (ELISA) were used to measure IFNγ in cell supernatant fluids. A bovine IFNγ kit (MABTECH, Cincinnati, OH) and a human IFNγ kit (Biolegend ELISA Max) were used to perform ELISAs, according to the manufacturer’s instructions. All measurements were performed in duplicate or triplicate.

To measure IFNγ production by flow cytometry, leukocytes were isolated as described above. Cells were treated with brefeldin A and incubated for 6 hrs at 37°C and 10% CO_2_. Bovine and human lymphocytes were stained as described above. Cells were then fixed with 2% paraformaldehyde for at least 10 min, washed once with PBS +2% horse serum, and then washed once with 0.2% saponin (Sigma) in PBS +2% horse serum. Bovine IFNγ was detected using a PE-conjugated mouse IgG1 mAb against bovine IFNγ (MCA1783E, ABD Serotec Inc., Raleigh, NC), whereas human IFNγ was detected using a PE-conjugated mouse IgG1 mAb (clone 4S.B3, Biolegend). For isotype controls, cells were stained with a PE-conjugated mouse IgG1 antibody (Biolegend). IFNγ antibodies and isotype controls were resuspended in 0.2% saponin. Cells were stained for 20 min at room temperature. After staining, cells were washed, then analyzed using a FACSCalibur with HTS.

**Figure 6:**
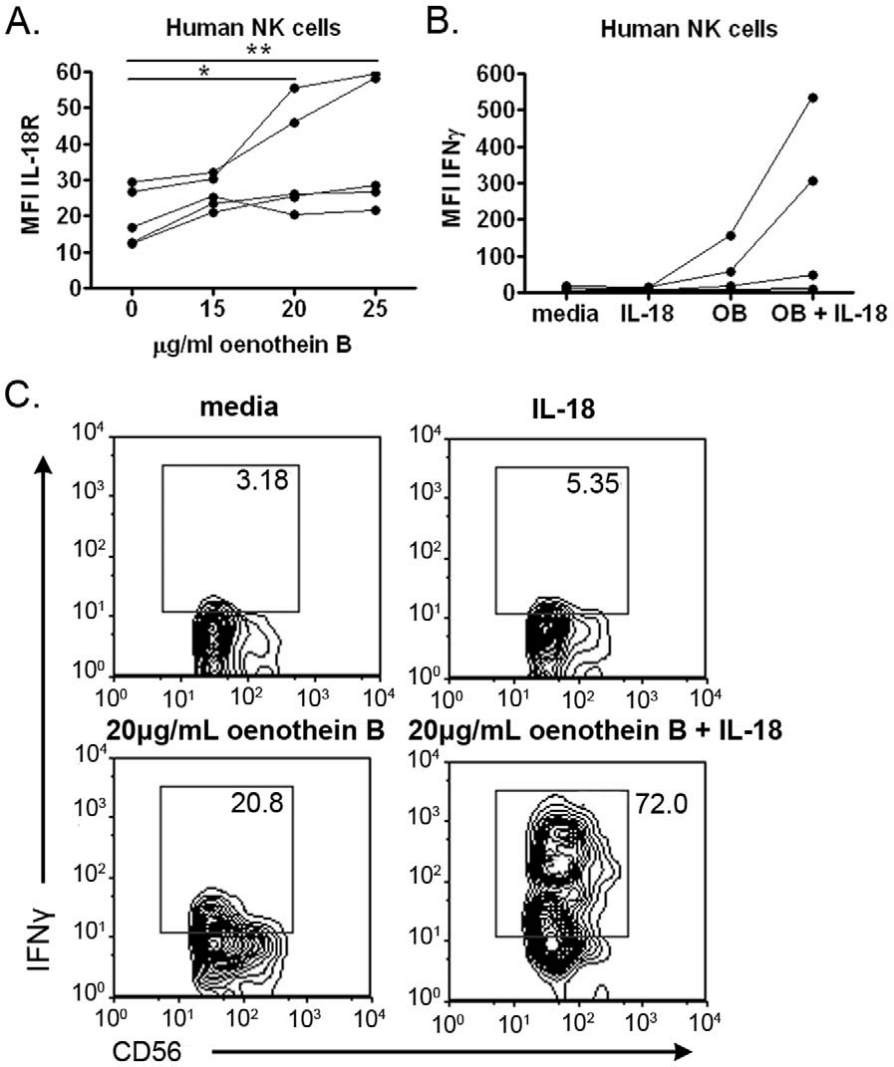
Priming of human NK cells to IL-18 by oenothein B. (A) Human PBMCs (10^5^ cells/well) were treated with the indicated concentrations of oenothein B or X-VIVO medium alone for 48 hrs, and expression of IL-18R on human NK cells was determined by flow cytometry. Significance was determined by One-way ANOVA with Bonferroni post-test. *p<0.05, **p<0.01 (B) Human PBMCs (10^5^ cells/well) were treated with 20 µg/ml oenothein B or X-VIVO medium alone for 48 hrs. Cells were washed with PBS and treated with 50 ng/ml rhu IL-18 for 6 hrs in the presence of brefeldin A. IFNγ staining [mean fluorescent intensity (MFI)] on human NK cells was determined by flow cytometry. The graphs represent data from five individuals, with each treatment analyzed in triplicate. (C) Representative examples of two-color flow cytometry plots comparing IFNγ staining on gated human NK cells treated with 20 µg/ml oenothein B, 50 ng/ml rhuIL-18, both, or X-VIVO medium alone. *p<0.05, **p<0.01, ***p<0.001.

**Figure 7:**
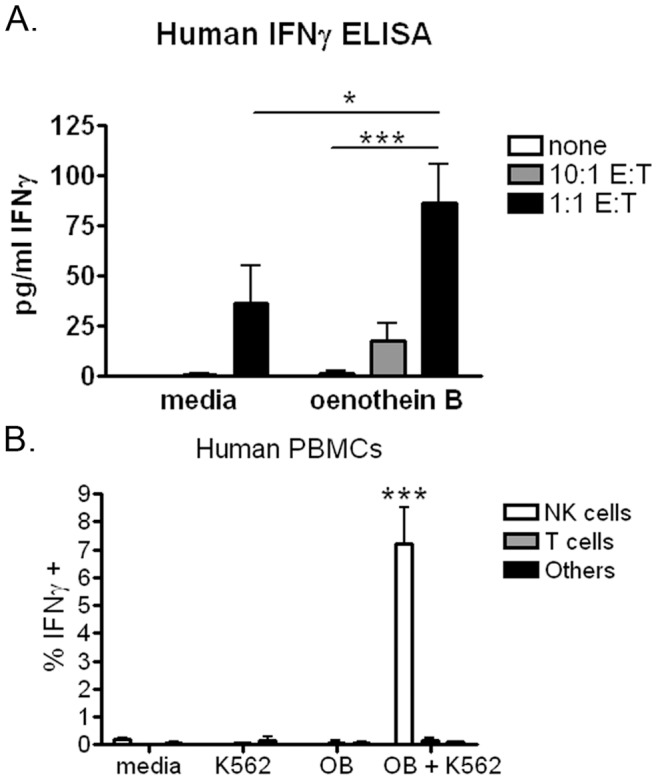
Priming of human NK cells to K562 cells by oenothein B. (A) Human PBMCs (10^5^ cells/well) were treated with 20 µg/ml oenothein B or X-VIVO medium alone for approximately 24 hrs. Cells were then washed and co-cultured with or without K562 cells at effector:target (E:T) ratios of 10∶1 and 1∶1 for approximately 42 hrs. After incubation, soluble IFNγ levels were measured by ELISA. The data represent pooled results from three donors and are expressed as mean +/− SEM. Samples were analyzed in duplicate. Statistical significance was measured by Two-way ANOVA with Bonferroni post-test. (B) Human PBMCs (10^5^ cells/well) were treated with 20 µg/ml oenothein B or X-VIVO medium alone for approximately 24 hrs. Cells were then washed and co-cultured with or without K562 cells at an effector:target (E:T) ratio of 1∶1 for approximately 18 hrs. After incubation, brefeldin A was added to the culture for 6 hrs. IFNγ expression by NK cells (CD3−/CD56+), T cells (CD3+), and others (CD3−/CD56-) was then measured by intracellular flow cytometry. The data represent pooled results from two donors and are expressed as mean +/− SEM. Samples were analyzed in duplicate. Statistical significance was measured by One-way ANOVA with Bonferroni post-test. *p<0.05, **p<0.01, ***p<0.001.

### Statistical Analysis

Statistical analyses were performed using Prism 4 (GraphPad Software, San Diego, CA). The data were analyzed by Student’s paired t-test, Student’s unpaired t-test, One-way ANOVA, or Two-way ANOVA as indicated.

## Results and Discussion

### Oenothein B Activates Human and Bovine Lymphocytes

Previously, we and others have found bovine PBMCs to be a useful model for the testing of novel innate lymphocyte agonists [4], [33]. The bovine model has also been used to study infections by *Mycobacterium* species and *Salmonella* species since it better reflects human diseases than rodent models [34]–[36]. To determine if oenothein B stimulated lymphocytes, we first evaluated IL-2Rα expression as a marker for activation of bovine PBMCs. IL-2Rα was upregulated on both bovine γδ T cells and NK cells after stimulation with oenothein B (20–40 µg/ml) for 24 hours *in vitro* (Figure 1A and Figure S1). Doses and timepoints were based upon preliminary dose and kinetic analyses (data not shown). We then examined if similar responses were seen in human PBMCs, using CD69 expression as a marker for activation. In these studies, oenothein B stimulation for 2 days *in vitro* induced CD69 expression on human CD3+ T cells, γδ T cells, CD8+ T cells, and CD3- CD56+ NK cells (Figure 1B and Figure S1) at similar doses known to stimulate monocytes [7]. Within the human γδ T cell population, both Vδ2+ (major circulatory subset) and Vδ2- (mainly Vδ1+ cells [37]) subsets were activated by oenothein B (Figure 1B), which is similar to responses induced by OPCs [4]. In addition, we also examined CD25 expression on human PBMCs. Interestingly, oenothein B stimulation induced CD25 expression on T cells, but not NK cells (Figure 2).

**Figure 8:**
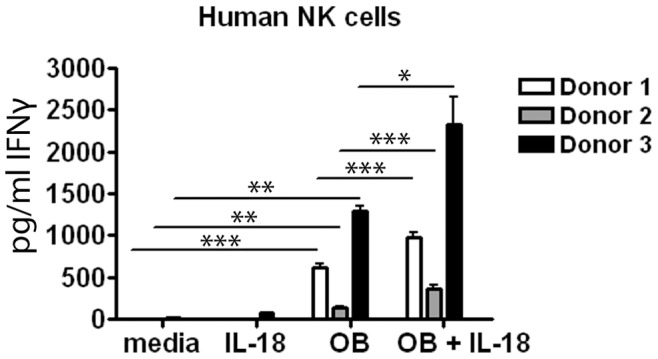
Oenothein B directly primes purified human NK cells to produce IFNγ. Human NK cells (5×10^4^ cells/well) were sorted and treated with 20 µg/ml oenothein B, 100 ng/ml rhu IL-18, both, or medium alone. After 24 hrs, soluble IFNγ was measured by ELISA. The graph represents soluble IFNγ levels in culture supernatant fluids from three separate experiments with three different donors. Error bars indicate SEM. Each sample was analyzed in triplicate. Significance was determined by One-way ANOVA with Bonferroni post-test. *p<0.05, **p<0.01, ***p<0.001.

### Oenothein B Primes Bovine PBMCs to Respond to IL-18

To examine the effects of oenothein B on IFNγ production in the bovine model, bovine PBMCs were treated with oenothein B for two days and secreted IFNγ was measured by ELISA. Similar to our studies on OPCs, we did not find significant amounts of IFNγ produced by oenothein B-treated bovine PBMCs (data not shown). However, in our original studies with OPCs, we found that OPC-treated γδ T cells had enhanced responses to secondary signals, such as IL-2 and TCR agonists [4]. In addition, others have found that feeding bovine calves polyphenols from pomegranate can enhance mitogen-induced IFNγ production by PBMCs [38]. Therefore, we hypothesized that oenothein B might enhance or prime responses to an inducer of IFNγ.

As such, we tested if oenothein B treatment of bovine lymphocytes enhanced responses to the IFNγ-inducing cytokine, IL-18. We also tested several well-studied polyphenols, epigallocatechin gallate (EGCG), resveratrol, curcumin, and theaflavin digallate (TFDG), all of which are potent antioxidents, to determine if such a response was a common property of polyphenols. When oenothein B–treated cells were subsequently treated with suboptimal doses of IL-18, IFNγ production was greatly enhanced compared to IL-18 or oenothein B alone (Figure 3). These data suggested that oenothein B could prime immune cells for enhanced IFNγ production in response to low doses of IL-18. Resveratrol and curcumin did not enhance IFNγ production in response to IL-18, but rather appeared to suppress the response, which would be consistent with previous studies describing their immunosuppressive properties [39], [40]. Both EGCG and TFDG enhanced IFNγ production in response to IL-18 in one of the calves tested, but their effect was not as consistent or as robust as oenothein B. The level of priming by oenothein B and the amount of IFNγ produced varied between animals. It is likely that these observed differences between the three calves were due to animal-specific responses to oenothein B, as our preliminary studies with IL-2Rα suggested that PBMCs from individual calves can respond differently to oenothein B. Based on these results, we focused our subsequent studies on oenothein B and its effect on IFNγ production.

### Presence of CD335+ Cells is Essential for Oenothein B Priming to IL-18

After observing enhanced IFNγ production by bovine cells pre-treated with oenothein B, we then determined which cells were important for this response. Since oenothein B has been shown to be a potent monocyte agonist, we first examined if these cells were essential for the priming responses. Monocytes were removed by flow cytometric sorting, and the priming response was again evaluated. Priming responses were still observed in monocyte-depleted PBMCs, although the level of priming was reduced in two out of three experiments (Figure S2). These results suggested that monocytes likely contributed to the response in the mixed population, but were not required for the response.

We then examined the importance of NKp46+ cells, since they are a major source of IFNγ induced by IL-12 and IL-18 in bovine lymphocytes [41]. NKp46, also known as CD335, is a NK cell marker, although it is expressed by other minor leukocyte-populations, including some γδ T cells [41]. To test the importance of these cells, we depleted cells expressing CD335 from bovine PBMCs and found that nearly all of the oenothein B-induced IFNγ priming response was absent compared to un-depleted PBMCs (Figure 4A).

Because CD335 is expressed on some γδ T cells [41], we examined whether γδ T cells contributed to the oenothein B-induced IFNγ response. Removal of γδ T cells reduced, but did not eliminate, the priming response (Figure S2). This result suggested that, like monocytes, γδ T cells contributed to, but were not required for, the response and further suggested that γδ TCR−/CD335+ cells were the primary source of IFNγ in these assays. As a final approach to confirm these results, multi-color intracellular cytokine analyses were performed. As shown in Figure 4, oenothein B-primed, IL-18-treated CD335+ cells expressed IFNγ (Figure 4B and 4C). The percentage of CD335+ cells was also enhanced by oenothein B (Figure 4C). However, this was likely due to activated monocytes adhering to the sample plates and being removed from the CD335- population rather than an expansion of CD335+ cells. Collectively, these data indicate that CD335+ NK cells are the major source of IFNγ produced in response to oenothein B and IL-18 in the bovine system.

### Oenothein B Induces IFNγ Production by Human Innate Lymphocytes

In our previous studies with oenothein B, we showed that treatment of human PBMCs with oenothein B promoted some IFNγ production, in contrast to the response we observed with bovine cells [7]. However, potential sources of this cytokine were not identified. We first confirmed our previous results in human PBMCs by cytokine ELISA (Figure 5A). We then examined if lymphocytes were a source of this induced IFNγ. Human T cells, including γδ T cells and CD8+ T cells, produced IFNγ in response to oenothein B (Figure 5B and 5C). The percentages of NK cells positive for IFNγ staining also increased in three out of five donors tested (Figure 5B), but minimal staining was observed compared to that seen in T cells (Figure 5C). Thus, unlike the bovine system, oenothein B-induced IFNγ production was not restricted to the human NK cell population.

In the bovine system, NK cells were primed to produce enhanced IFNγ in response to IL-18. We tested whether the same could be true for human NK cells. First, multi-color flow cytometry showed that IL-18 receptor was increased on oenothein B-treated human NK cells in two out of five donors (Figure 6A). Similarly, further analyses showed that in two, possibly three, of five human PBMC preparations, IFNγ production was increased in oenothein B-primed, IL-18-treated human NK cells compared to cells treated with oenothein B or IL-18 alone (Figure 6B and 6C).

We then tested if oenothein B could enhance IFNγ production in response to the NK cell target leukemic cell line, K562. Others have shown that stimulation by K562 cells induces IFNγ secretion by NK cells and that this response can be enhanced by the presence of a second stimuli [42]. Consistent with these reports, pretreatment of human PBMCs with oenothein B enhanced IFNγ production in response to K562 cells compared to untreated PBMCs (Figure 7A) and NK cells were the major cell population primed by oenothein B for enhanced IFNγ production (Figure 7B).

NK cell numbers and activity can vary significantly from donor to donor. To address whether the inconsistency seen in PBMC preparations in response to oenothein B and IL-18 might be due to variable numbers of NK cells between PBMC samples or variable influences by other cells within the mixed populations on the NK cells, human NK cells were sorted, and then equal cell numbers were treated with oenothein B alone, IL-18 alone, or a combination of both. IFNγ production was measured 24 hrs later by ELISA. As shown in Figure 8, oenothein B alone directly induced IFNγ production by NK cells and there was an increase in IFNγ production with the combined treatment in all donors tested, although the amount of IFNγ produced varied between donors. This variability in IFNγ production by NK cells has been observed in other studies and may have a genetic component [42], [43]. These data further suggest that, as with bovine cells, oenothein B treatment has the potential to augment IFNγ production by human NK cells alone and in response to IL-18. In addition, these data suggest that oenothein B can directly prime these cells to respond to IL-18.

Collectively, our results show that, in addition to monocytes, oenothein B stimulates subsets of bovine and human lymphocytes, including NK cells, CD8+ T cells, and both Vδ2+ and Vδ2- γδ T cells, by upregulating IL-2Rα and/or CD69 on these cells. We also show that oenothein B promotes the production of IFNγ by human lymphocytes, specifically γδ T cells and CD8+ T cells. Furthermore, we demonstrate that IFNγ production by NK cells can also be induced by oenothein B, although this response was not as robust or consistent as that seen in T cells. Interestingly, differences in the capacity of oenothein B to induce IFNγ production by T cells was observed between human and bovine cells, as oenothein B alone did not directly induce significant IFNγ secretion by bovine T cells as it did with human T cells. These data suggest that certain polyphenols may exert species-specific effects and that immunomodulatory effects of polyphenols demonstrated in one species may not always be conserved in other species. Thus, analysis of the immunomodulating properties of polyphenols cannot rely solely on animal testing, and a combination of animal and human cell testing is required to identify relevant, conserved responses.

A possible explanation for some of the differences observed between human and bovine T cells in these studies could be due to differences in ages, as young calves were used for our bovine studies while adults were used for our human studies. It has been shown that IFNγ secretion by T cells can increase with age, correlating with an increase in CD45RO+ T cells [44]. Therefore, future studies could examine the effects of aging on these responses. It is possible that lymphocyte responses to certain polyphenols in young bovine calves are more reflective of those that might occur in children, suggesting a potential new use for this animal model in the study of the effects of dietary polyphenols on neonatal and adult lymphocytes.

A potentially important and conserved response to oenothein B is enhanced IFNγ secretion following exposure to suboptimal IL-18 concentrations, which was observed in both bovine and human NK cells. The synergistic effect of oenothein B and IL-18 for enhancing IFNγ production by NK cells was observed in mixed PBMC cultures, NK cell-depleted PBMCs, as well as sorted NK cells. Our earlier studies demonstrated that oenothein B could induce IL-12 production by monocytes [7], which others have found synergizes with IL-18 to produce IFNγ [45]. Thus, this could provide an explanation for oenothein B’s ability to enhance IL-18-induced IFNγ production in some of our experiments; however, the enhanced production of IFNγ observed in sorted NK cell cultures suggests a direct effect on NK cells by oenothein B. Additionally, oenothein B enhanced IFNγ secretion in response to an NK cell target cell line, suggesting that the ability of oenothein B to enhance IFNγ secretion is not restricted to IL-18, but also occurs upon co-culture with tumor cell targets.

In conclusion, our results expand upon previous studies suggesting that oenothein B stimulates innate and antitumor immunity, and further characterizes this activity, suggesting that lymphocyte activation and IFNγ production may contribute to these responses. The production of IFNγ by lymphocytes and other cells enhances antitumor immunity by a number of mechanisms, and it will be important to examine whether lymphocytes and/or IFNγ play an important role in the antitumor properties of oenothein B *in vivo*. In addition, IFNγ production is a vital step in the host defense against numerous pathogens, including viruses and intracellular bacteria. Therefore, our data also suggest a potential mechanism whereby oenothein B could enhance antiviral and antibacterial immunity *in vivo*. Thus, it will also be important to examine if oenothein B enhances host defense against various pathogens whose clearance relies on lymphocyte activity and IFNγ production. Further work is also necessary to identify the receptors and signaling pathways involved in these immune stimulatory effects of oenothein B. Finally, these studies suggest that oenothein B may be a promising candidate for therapeutic development to supplement immunotherapies, especially those involving IL-18.

## Supporting Information

Figure S1 **Oenothein B induces IL-2Rα or CD69 on bovine and human NK cells.** (A) Bovine PBMCs (10^5^ cells/well) were treated with 20 µg/ml oenothein B in X-VIVO medium for 24 hrs, and IL-2Rα expression on NK cells was measured by multi-color flow cytometry. Representative examples of two-color flow cytometry plots comparing IL-2Rα staining on oenothein B-treated and untreated bovine NK cells (CD335+) from each animal are shown. (B) Human PBMCs (10^5^ cells/well) were treated with 40 µg/ml oenothein B in cRPMI medium for 48 hrs. CD69 expression on NK cells was then measured by flow cytometry. Representative examples of two-color flow cytometry plots comparing CD69 staining on oenothein B-treated and untreated human NK cells from each donor are shown.
(TIF)

Figure S2 **Effect of monocyte and γδ T cell** **depletion on oenothein B-priming of bovine PBMCs.** Bovine PBMCs (10^5^ cells/well) were depleted of (A) monocytes or (B) γδ T cells and treated with 20 µg/ml oenothein B or X-VIVO medium alone for 24 hrs. Cells were then washed and treated with 10 ng/ml rhu IL-18 or medium alone for 18 hrs. After incubation, IFNγ levels in the supernatant fluids were measured by ELISA. The data are expressed as mean +/− SEM of three independent experiments comparing depleted PBMCs to un-depleted controls tested concurrently. All samples were tested in duplicate or triplicate. Statistical significance was measured by Two-way ANOVA with Bonferroni post-test. *p<0.05, **p<0.01, ***p<0.001
(TIF)
