# Supplementary figures and images for: Oenothein B, a Cyclic Dimeric Ellagitannin Isolated from Epilobium angustifolium, Enhances IFNγ Production by Lymphocytes
Source: PLoS One. 2012 Nov 30;7(11):e50546. doi: 10.1371/journal.pone.0050546 (PMC3511557; doi:10.1371/journal.pone.0050546)

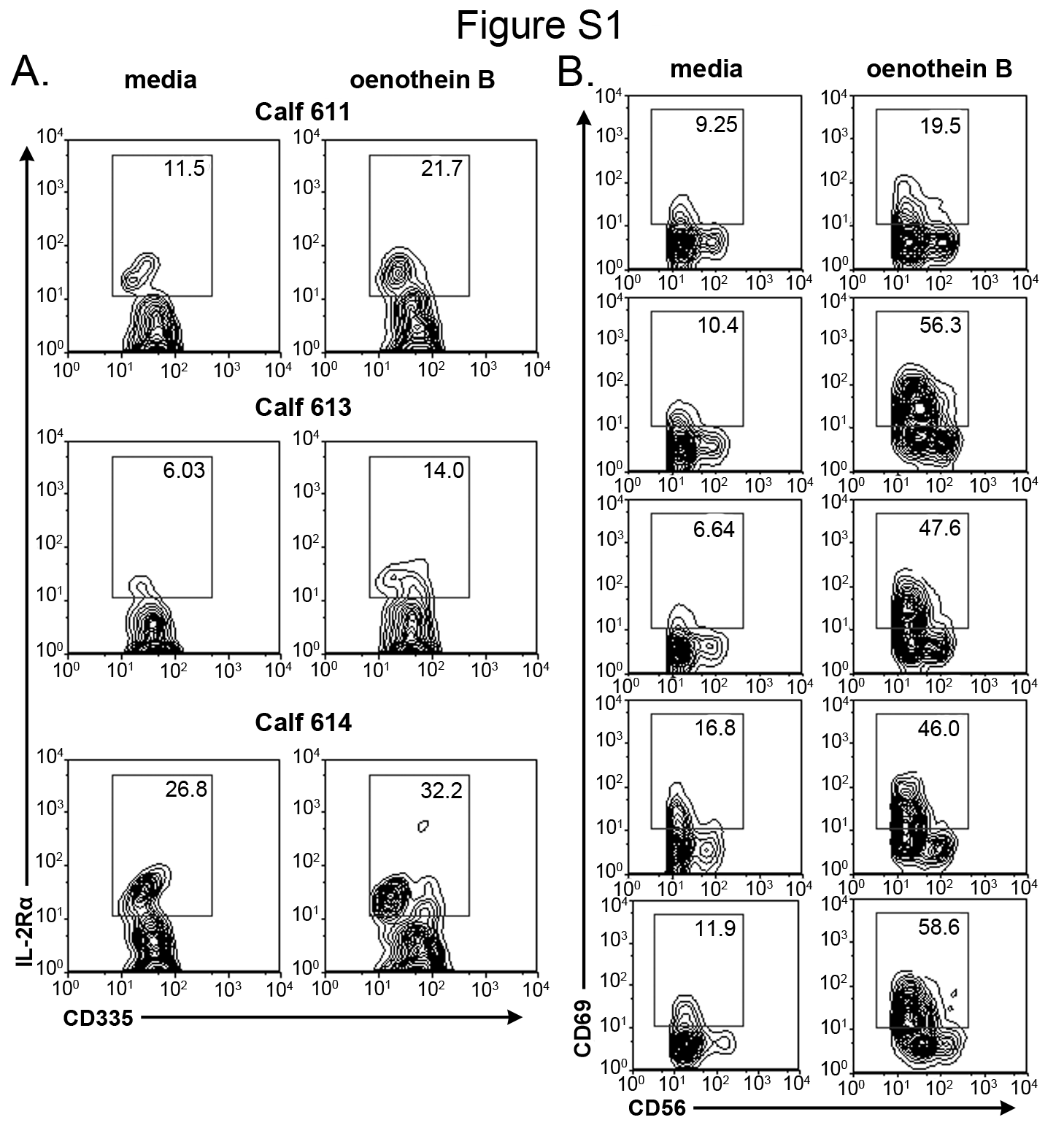

Supplement: Figure S1 — Oenothein B induces IL-2Rα or CD69 on bovine and human NK cells. (A) Bovine PBMCs (105 cells/well) were treated with 20 µg/ml oenothein B in X-VIVO medium for 24 hrs, and IL-2Rα expression on NK cells was measured by multi-color flow cytometry. Representative examples of two-color flow cytometry plots comparing IL-2Rα staining on oenothein B-treated and untreated bovine NK cells (CD335+) from each animal are shown. (B) Human PBMCs (105 cells/well) were treated with 40 µg/ml oenothein B in cRPMI medium for 48 hrs. CD69 expression on NK cells was then measured by flow cytometry. Representative examples of two-color flow cytometry plots comparing CD69 staining on oenothein B-treated and untreated human NK cells from each donor are shown. (TIF) [file pone.0050546.s001.tif]

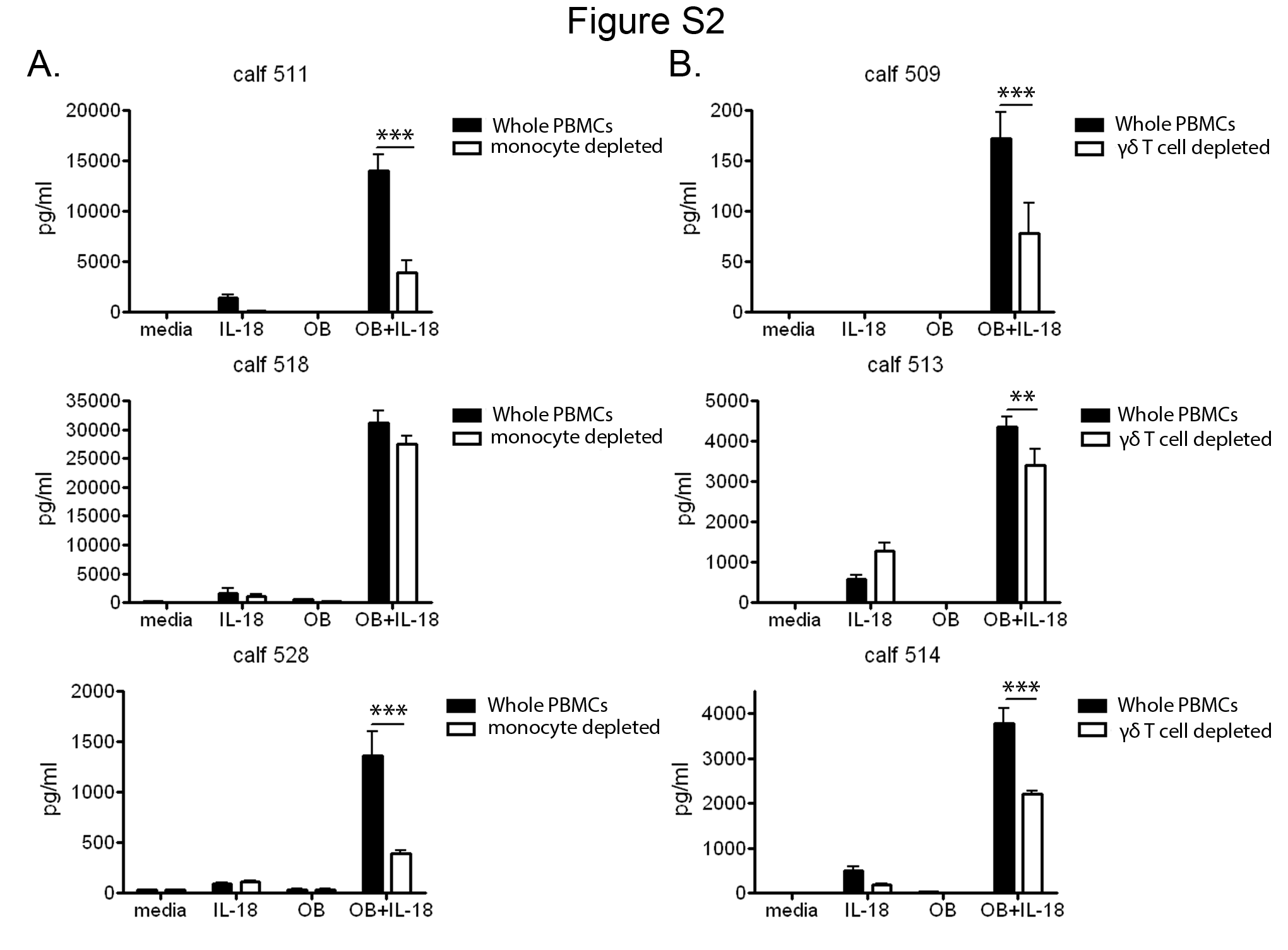

Supplement: Figure S2 — Effect of monocyte and γδ T cell depletion on oenothein B-priming of bovine PBMCs. Bovine PBMCs (105 cells/well) were depleted of (A) monocytes or (B) γδ T cells and treated with 20 µg/ml oenothein B or X-VIVO medium alone for 24 hrs. Cells were then washed and treated with 10 ng/ml rhu IL-18 or medium alone for 18 hrs. After incubation, IFNγ levels in the supernatant fluids were measured by ELISA. The data are expressed as mean +/− SEM of three independent experiments comparing depleted PBMCs to un-depleted controls tested concurrently. All samples were tested in duplicate or triplicate. Statistical significance was measured by Two-way ANOVA with Bonferroni post-test. *p<0.05, **p<0.01, ***p<0.001 (TIF) [file pone.0050546.s002.tif]
